# Mesoporous halloysite nanotubes modified by CuFe_2_O_4_ spinel ferrite nanoparticles and study of its application as a novel and efficient heterogeneous catalyst in the synthesis of pyrazolopyridine derivatives

**DOI:** 10.1038/s41598-019-42126-9

**Published:** 2019-04-03

**Authors:** Ali Maleki, Zoleikha Hajizadeh, Peyman Salehi

**Affiliations:** 1 Catalysts and Organic Synthesis Research Laboratory, Department of Chemistry, Iran University of Science and Technology, Tehran, 16846-13114 Iran; 2 Department of Phytochemistry, Medicinal Plants and Drugs Research Institute, Shahid Beheshti University, Evin, Tehran, Iran

## Abstract

In this study, mesoporous halloysite nanotubes (HNTs) were modified by CuFe_2_O_4_ nanoparticles for the first time. The morphology, porosity and chemistry of the CuFe_2_O_4_@HNTs nanocomposite were fully characterized by Fourier transform infrared (FT-IR) spectroscopy, field-emission scanning electron microscopy (FE-SEM) image, transmission electron microscope (TEM) images, energy-dispersive X-ray (EDX), X-ray diffraction (XRD) pattern, Brunauer-Emmett-Teller (BET) adsorption-desorption isotherm, thermogravimetric (TG) and vibrating sample magnetometer (VSM) curve analyses. The results confirmed that CuFe_2_O_4_ with tetragonal structure, uniform distribution, and less agglomeration was located at HNTs. CuFe_2_O_4_@HNTs nanocomposite special features were high thermal stability, crystalline structure, and respectable magnetic property. SEM and TEM results showed the nanotube structure and confirmed the stability of basic tube in the synthetic process. Also, inner diameters of tubes were increased in calcination temperature at 500 °C. A good magnetic property of CuFe_2_O_4_@HNTs led to use it as a heterogeneous catalyst in the synthesis of pyrazolopyridine derivatives. High efficiency, green media, mild reaction conditions and easily recovery of the nanocatalyst are some advantages of this protocol.

## Introduction

Efforts to achieve the benefits of both heterogeneous and homogeneous catalysts, caused suggestion of magnetic nanoparticles (MNPs) by scientists^1^. Due to their unique features such as reusability, low toxicity, large surface area, ease of separation and low cost, they are applied in different industries and research fields^2^. Spinel ferrites with the general formula MFe_2_O_4_ (M: Mn, Co, Ni, Cu, Mg, etc.) exhibited more catalytic activities compare to single-component metal oxides^3^. Among the different ferrites, CuFe_2_O_4_ with the mostly spinel structure is more attractive and applicable in different areas such as adsorption^4^, catalysis^5^, and gas sensors^6^. The surface of many kinds of NPs was modified and coated with available and eco-friendly substances such as chitosan, cellulose, and clay to overcome the agglomeration and improve the colloidal stability of MNPs^7–9^.

Halloysite is a group of aluminosilicate mineral with nanotubular structure^10,11^. HNTs with the external diameter of 40–60 nm and the internal diameter of 10–15 nm are classified as mesoporous materials^12^. The structure and morphology of HNTs are dependent on the pore properties of nanotubes, also, it’s size could be easily changed under thermal, acidic and alkaline treatments^13^. The nano-sized tubular structure, mesoporosity and macroporosity of HNTs have been applied in several areas such as environmental uses^14^, tissue engineering^15^, cosmetic^16^, medicine^17^ and catalysis^18^. HNTs with two kinds of hydroxyl groups in the inside and outside walls can be modified by different functional groups. Recently, several studies have been carried out for deposition of MNPs onto the HNTs and the developed nanocomposites with enhanced abilities could be used in various applications^19,20^.

Multicomponent reactions (MCRs) with features like atom economy, experimental simplicity, synthetic efficiency and formation of several bonds in one unit operation have been proposed in the synthesis of biologically-active organic compounds particularly in the case of heterocycles such as pyrazolopyridines^21^. Pyrazole derivatives are one of the most important nitrogen-containing heterocyclic compounds with antileishmanial^22^, antimicrobial^23^ and antiviral^24^ properties. Due to these biological and pharmacological properties, synthesis of pyrazoles has received considerable attention in chemistry. Several methods have been reported for the synthesis of pyrazolopyridines in the presence of diverse catalysts such as L-proline in [bmim]BF_4_ ^25^, acetic acid^26^, CuCr_2_O_4_ NPs^27^ and cellulose-based magnetic nanocomposite^28^. Most reports have some disadvantages like using non-reusable and toxic catalysts, harsh reaction conditions, low yields of products and long reaction times. As a result, for the synthesis of heterocyclic compounds with more efficient procedures, improvement of existing methods by using the natural-based nanocatalyst has been emphasized.

**Figure 1:**
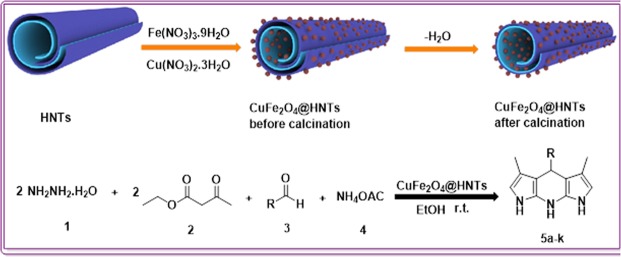
Synthesis of pyrazolopyridine derivatives (**5a-k**) in the presence of CuFe_2_O_4_@HNTs nanocatalyst.

From the above-mentioned viewpoints and according to previous scientific reports^29,30^, in this research, CuFe_2_O_4_@HNTs as a novel, eco-friendly and mesoporous magnetic nanocatalyst was designed, synthesized, characterized and applied in a one-pot multicomponent reaction as a heterogeneous catalyst. Synthesis of pyrazolopyridine derivatives (**5a-k**) starting from hydrazine hydrate, ethyl acetoacetate, aromatic aldehydes and ammonium acetate were achieved in high-to-excellent yields at ambient conditions (Fig. 1).

## Results and Discussion

In this research, CuFe_2_O_4_@HNTs as a mesoporous nanocomposite was synthesized by a simple method using eco-friendly materials. The properties of this novel and retrievable magnetic nanocomposite such as nanotube morphology, thermal stability, porosity, magnetic properties were examined by different analyses like FT-IR, FE-SEM, TEM, BET, EDX, XRD, TGA and VSM. The catalytic performance of CuFe_2_O_4_@HNTs was checked in the synthesis of pyrazolopyridines. The sustainable nanocatalyst can be easily reused after 8 runs.

### Characterization of the prepared CuFe_2_O_4_@HNTs nanocatalyst

**Figure 2:**
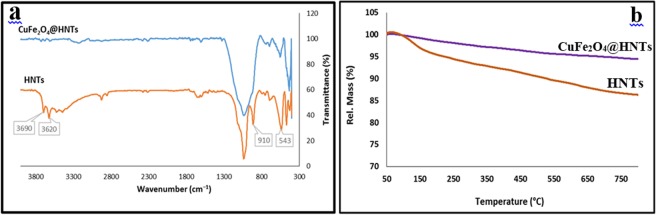
(**a**) FT-IR spectra and (**b**) TG curves of HNTs and CuFe_2_O_4_@HNTs.

FT-IR spectroscopy as a primary method was performed to indicate the formation of CuFe_2_O_4_@HNTs nanocomposite (Fig. 2). As can be seen, the absorption bands at 543 and 470 cm^−1^ were assigned to the bending vibration of Al-O-Si and Si-O-Si, respectively. The absorption band at 910 cm^−1^ is related to deformation of inner hydroxyl groups of Al–O–H, also, the absorption band at 1114 cm^−1^ is ascribed to the Al-OH bending vibration. Si–O stretching band was appeared at 1029 cm^−1^. Other characteristic absorption bands at 3690 and 3620 cm^−1^ are attributed to the stretching vibration of Al-OH bonds^31^. After calcination and synthesis of the nanocomposite because of dihydroxylation process, the characteristic absorption bands of Al-OH bond disappeared and other absorption bands to be broad gentle^32^. The characteristic bands of Cu-O and Fe-O at 540 cm^−1^ are overlapped with the bending vibration of Al-O-Si^5^.

The thermal stability of the synthesised nanocomposite was studied by TGA analysis in the air atmosphere and heating rates at 10 °C/min, over the temperature range of 50–800 °C (Fig. 2b). As can be seen in HNTs TGA curve, the losing weight of HNTs was about 20% between 50 to 800 °C and is related to molecules which are physically absorbed on to the surface of HNTs and loss of hydroxyl groups. The thermal stability of HNTs was improved in the presence of CuFe_2_O_4_ NPs. CuFe_2_O_4_@HNTs nanocomposite doesn’t decompose easily and at high temperatures showed a little weight loss.

**Figure 3:**
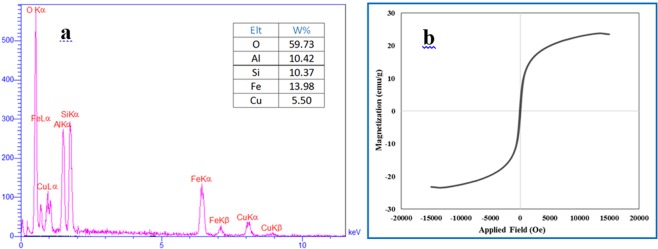
(**a**) EDX analysis and (**b**) VSM magnetization curve of CuFe_2_O_4_@HNTs nanocomposite.

EDX was applied to identify the elements of CuFe_2_O_4_@HNTs nanocomposite. The nanocatalyst included different elements such as iron, copper, oxygen, aluminum and silicon. The result of the EDX analysis shown in Fig. 3a, confirms the presence of all elements at different percentages.

The magnetic recovery of a nanocomposite is one of its most important properties. In the previously reported study, the magnetization of neat CuFe_2_O_4_ NPs was around 45 electromagnetic units per gram (emu g^−1^)^33^. As displayed by VSM analysis (Fig. 3b), the saturation magnetization values of CuFe_2_O_4_@HNTs nanocomposite was around 30 emu g^−1^. The loading of CuFe_2_O_4_ NPs on HNTs causes the saturation magnetization level to decrease and confirms the synthesis of the nanocomposite. However, this magnetic property is acceptable and nanocomposite can be easily separated from the reaction mixture.

**Figure 4:**
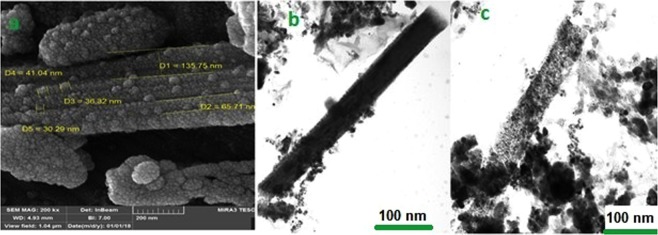
(**a**) FE**-**SEM image and (**b,c**) TEM images of CuFe_2_O_4_@HNTs nanocomposite.

The size distribution and the morphology of the nanocomposite were examined by FE-SEM image analysis. As can be seen in Fig. 4a, the structure of the basic tubes of HNTs were stable but the inner diameters of tubes were increased during the synthesis of the nanocomposite at 500 °C and have the capacity for greater loading of CuFe_2_O_4_ NPs on the outer and inner surface of HNTs. Roughness and particles with regular shapes on the surface of nanotubes confirmed the synthesis of CuFe_2_O_4_ NPs. These MNPs were distributed with uniform size of 35.88 nm.

Also, TEM image analysis was used to reveal the MNPs location (Fig. 4b,c). The TEM images of the nanocomposite clearly showed the cylinder-shaped of the HNTs and loading of CuFe_2_O_4_ NPs in inside and outside of nanotubes in gray color.

**Figure 5:**
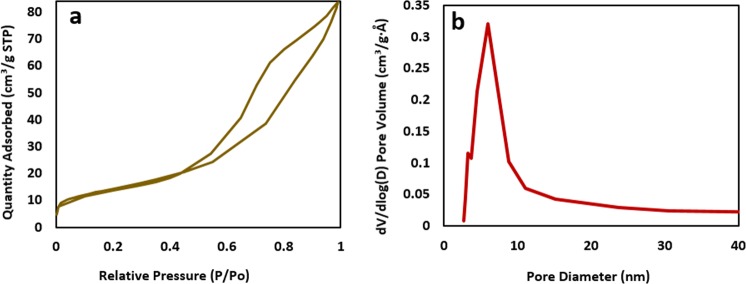
(**a**) N_2_ adsorption curve, (**b**) pore size distribution curve of CuFe_2_O_4_@HNTs nanocomposite.

The surface area, pore volume and pore size properties of CuFe_2_O_4_@HNTs nanocomposite were determined by nitrogen adsorption and desorption analyses. Adsorption isotherm is shown in Fig. 5a. As can be seen, this isotherm could be categorized to the class of type IV as a mesoporous material l according to IUPAC classification^34^. As stated in reported articles, BET surface area of HNTs is around 47.8 m^2^g^−1^, while, CuFe_2_O_4_@HNTs nanocomposite show a slight increase to 51.13 m^2^g^−1^. This result confirmed the loading of CuFe_2_O_4_ on HNTs_._ Also, due to the placement of CuFe_2_O_4_ inside the HNTs, the pore volume of CuFe_2_O_4_@HNTs decreased to 0.126, by comparison with the reported pore volume^35^. In addition, owing to the calcination process, the pore size of CuFe_2_O_4_@HNTs increased to 9.87 (Fig. 5b).

**Figure 6:**
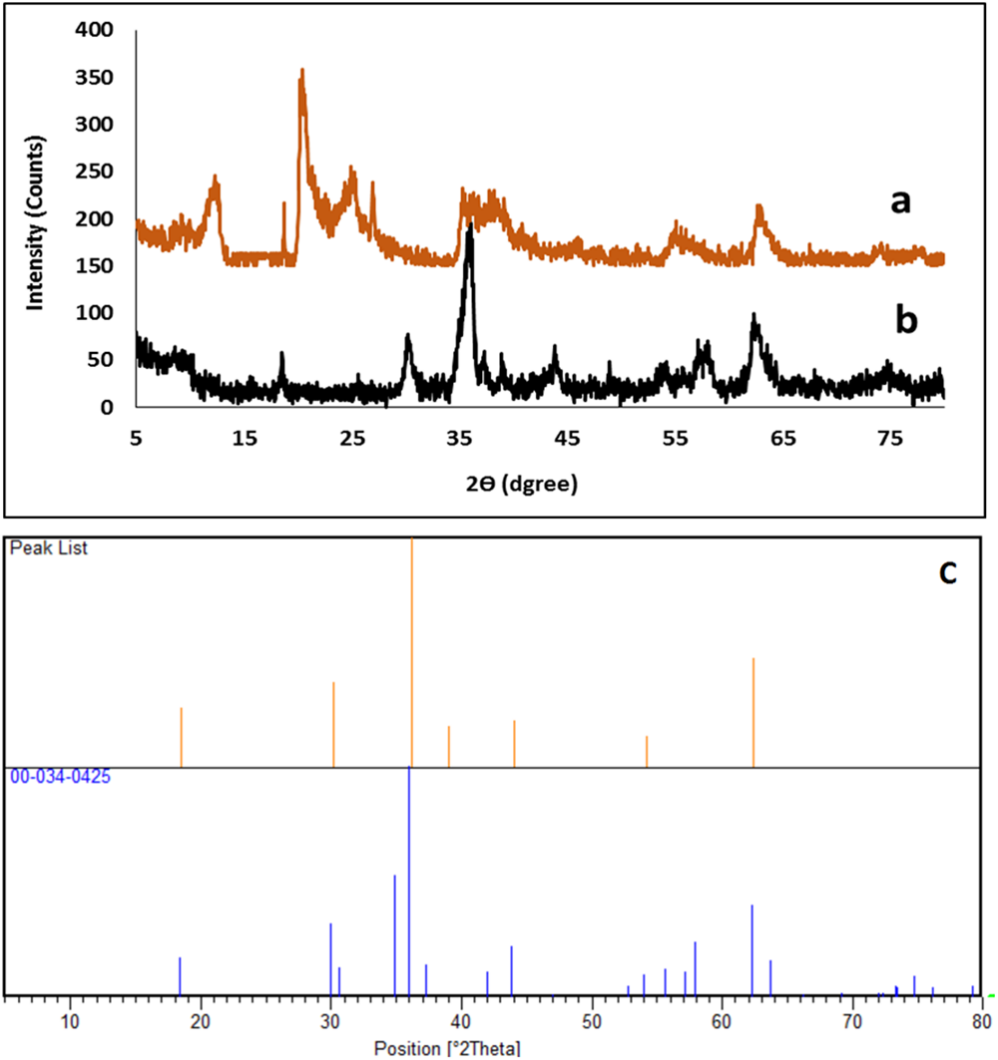
The XRD pattern of: (**a**) HNTs, (**b**) CuFe_2_O_4_@HNTs, (**c**) the reference CuFe_2_O_4_.

In order to investigate the morphology and crystallinity of CuFe_2_O_4_@HNTs, XRD pattern was studied. As shown in Fig. 6b, CuFe_2_O_4_ NPs with tetragonal structure was loaded in inner and outer of HNTs. The diffraction angles (2θ) 18.59, 30.14, 36.12, 38.99, 44.00, 54.18 and 62.37 are fully complied with the characteristic data of (JCPDS card no. 00-034-0425). The typical diffraction peaks of HNTs appeared at 12.62° and 20.26° (Fig. 6a), but during the synthesis of nanocomposite and calcination in the temperature of 500 °C, crystalline structure of HNTs changed to amorphous structure and gentle peak in 25.61 appeared^35^.

### Catalytic application of CuFe_2_O_4_@HNTs nanocatalyst in the synthesis of pyrazolopyridine derivatives

The catalytic application of the nanocomposite has been investigated in the synthesis of pyrazolopyridine derivatives. In order to optimize the reaction condition, the four-component reaction of hydrazine hydrate **1** (2 mmol), ethyl acetoacetate **2** (2 mmol), benzaldehyde **3** (1 mmol), and ammonium acetate **4** (3 mmol) was chosen as the model reaction. Different catalysts, solvent and their ratios were studied. It was found that CuFe_2_O_4_@HNTs nanocomposite shows an excellent catalyst activity as compared with the single component of CuFe_2_O_4_ or HNTs. Apparently, the HNTs structure caused loading the MNPs inside and outside of the tubes and increasing the efficiency of the catalyst. Also, composite CuFe_2_O_4_ and HNT_S_ prevent the agglomeration of MNPs and improved the efficiency of the catalyst. A considerable yield was obtained in the presence of 50 mg of the catalyst. In addition, this procedure showed significant improvements in the time and yield. The results summarized in Table S1 in Supplementary Information File.

The efficiency of this protocol was examined by using a series of different aldehydes. All of the aromatic aldehydes having either electron-withdrawing or electrondonating substituents produced pyrazolopyridine derivatives (**5a-k**) in high-to-excellent yields. The results are summarized in Table 1.

**Table 1:** Synthesis of pyrazolopyridine derivatives **5a-k** in optimal conditions^a^.

Entry	R	Product	Yield^b^ (%)	Mp (°C)	
				Found	Reported
1	C_6_H_5_	**5a**	96	240–241	240–242^27^
2	3-NO_2_-C_6_H_4_	**5b**	95	285–287	286–288^37^
3	4-NO_2_-C_6_H_4_	**5c**	93	296–298	295–297^27^
4	4-CN-C_6_H_4_	**5d**	93	286–288	286–288^37^
5	3-Br-C_6_H_4_	**5e**	90	244–246	245–247^36^
6	4-Cl-C_6_H_4_	**5f**	94	255–257	255–257^27^
7	4-F-C_6_H_4_	**5g**	96	259–261	258–260^38^
8	4-OH	**5h**	90	268–270	269–271^38^
9	4-Me-C_6_H_4_	**5i**	93	244–246	245–247^38^
10	2,4-(Cl)_2_-C_6_H_3_	**5j**	91	308	>300^37^
11	pyrid-2-yl	**5k**	90	219–221	218^36^

**Figure 7:**
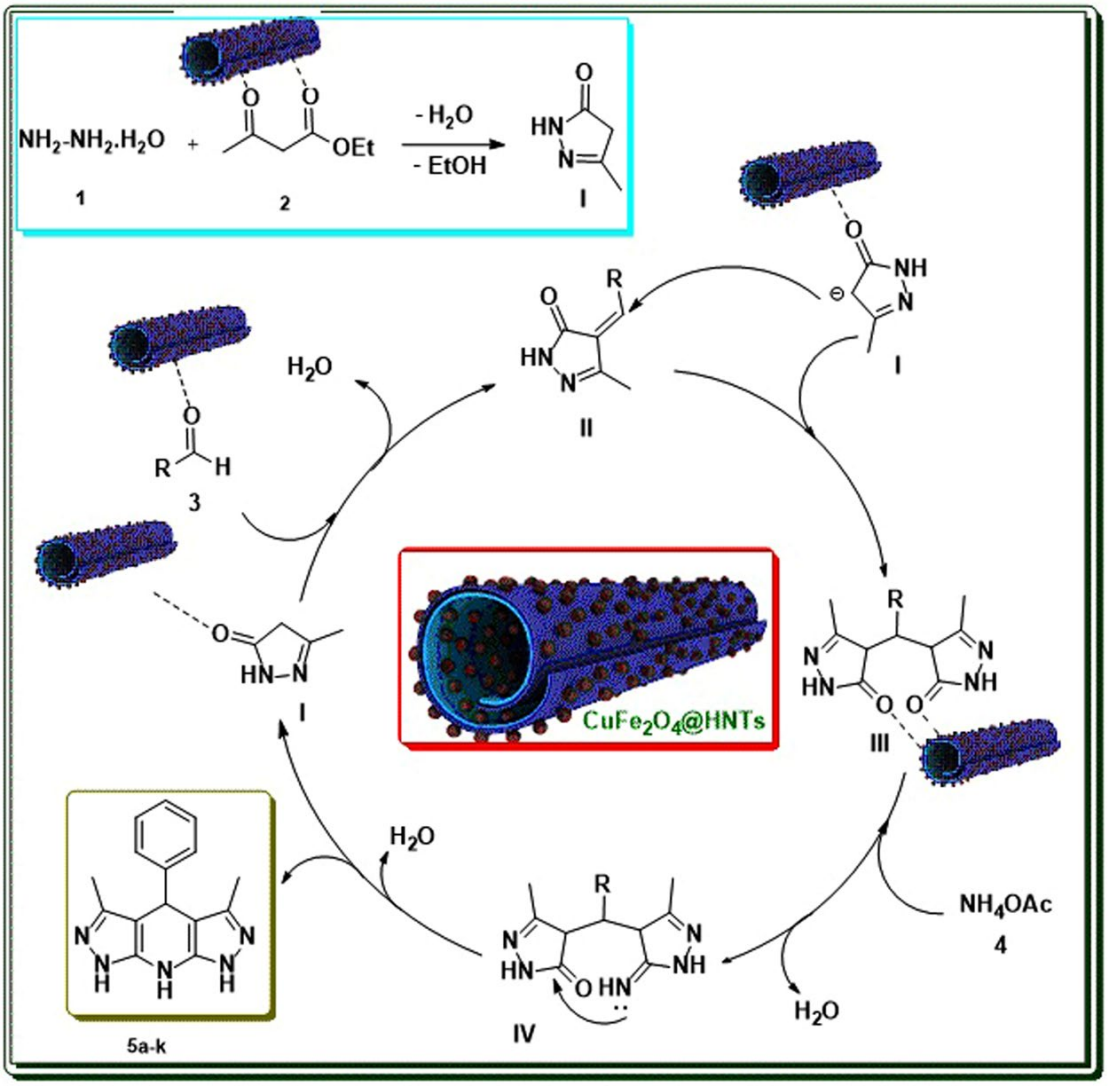
Proposed mechanism for the synthesis of **5a-k** by using CuFe_2_O_4_@HNTs.

A Hantzsch-type mechanism for the formation of pyrazolopyridine derivatives was proposed in Fig. 7. As can be seen, CuFe_2_O_4_@HNTs nanocomposite as a Lewis acid plays an important role in all parts of the reaction and accelerated the multicomponent process.

According to the reported article^36^, firstly ethyl acetoacetate was activated by CuFe_2_O_4_@HNTs and nucleophilic attack of hydrazine to the carbonyl group led to the pyrazolone ring (**I**) via cyclocondensation and removal of H_2_O and EtOH. Then, intermediate **II** was formed by a Knoevenagel condensation of activated aldehydes and pyrazolone ring. The reaction can be continued via Michael addition by attack of the second pyrazolone ring upon the intermediate **II** and the formation of intermediate **III**. Nucleophilic attack of ammonia on intermediate **III** in the presence of CuFe_2_O_4_@HNTs gave compound **IV**. Finally, intramolecular cyclization, dehydration and, tautomerization of compound **IV** provided **5a-k**.

### Reusability study of CuFe_2_O_4_@HNTs nanocomposite

Catalyst’s reusability is among the most significant factors in view of industrial and commercial aspects. For that reason, recoverability and reusability of CuFe_2_O_4_@HNTs nanocatalyst was explored. After the first test, the catalyst was recovered by the use of an external magnet from the model reaction. The recovered catalyst was washed several times with ethanol to remove any organic products and dried. Then, it was used again in the next runs under the same reaction conditions. Recyclability of the catalyst was evaluated for eight runs without significant decrease in activity (Fig. S5). Also, in order to investigate the stability of the recycled catalyst, it was identified by FT-IR and EDX analyses (see Figs S6 and S7 in Supporting Information File).

## Experimental

### General

Chemicals were purchased from Sigma-Aldrich and Merck companies and applied without any further purifications. Fourier transforms infrared (FT-IR) spectra were recorded on a Shimadzu IR-470 spectrometer by the method of KBr pellet. Melting points were measured on an Electrothermal 9100 apparatus. Field-emission scanning electron microscopy (FE-SEM) was performed by a MIRA 3 TESCAN microscope with an attached camera. Transmission electron microscopy (TEM) images were obtained by a Philips CM120 instrument. Brunauer-Emmett-Teller (BET) analysis was achieved by micromeritics ASAP 2020. Thermogravimetric analysis (TGA) was taken by Bahr-STA 504 instrument. X-ray diffraction (XRD) measurements were reported on a JEOL JDX–8030 (30 kV, 20 mA). Elemental analysis of the nanocatalyst was carried out by energy-dispersive X-ray (EDX) analysis recorded Numerix DXP-X10P. Lakeshore 7407 (Meghnatis Kavir Kashan Co., Iran) vibrating sample magnetometers (VSMs) was used for examining the magnetic measurement of the solid sample. ^1^H and ^13^C nuclear magnetic resonance (NMR) spectra were recorded on a Bruker DRX-300 Avance spectrometer at 300 and 75 MHz, respectively.

### Preparation of CuFe_2_O_4_@HNTs

0.477 g (1.17 mmol) of Fe(NO_3_)_3_.9H_2_O and 0.14 g (0.58 mmol) of Cu(NO_3_)_2_.3H_2_O dissolved in 20 mL distilled water. Then, 0.50 g of HNTs was added to metal ions solution and the mixture was stirred for 1 h at room temperature. To provide nanocomposite, 5 mL solution of NaOH (0.50 g, 12 mmol) was added dropwise during 10 min to the initial mixture at room temperature. The final mixture was warmed at 90 °C and stirred for 2 h. The black precipitate of CuFe_2_O_4_@HNTs was separated by an external magnet and washed 4 times with distilled water. The synthesized nanocomposite was dried in air oven at 80 °C for 4 h and kept in a furnace at 500 °C for 5 h.

### General procedure for the synthesis of pyrazolopyridine derivatives **5a–k**

A mixture of hydrazine hydrate **1** (2 mmol), ethyl acetoacetate **2** (2 mmol), aromatic aldehydes **3** (1 mmol), and ammonium acetate **4** (3 mmol), in the presence of CuFe_2_O_4_@HNTs nanocatalyst (5 mg) was reacted in ethanol at room temperature. After 20 min, completion of the reaction was screened by thin layer chromatography (TLC). Then, the catalyst was separated simply by an external magnet. By addition of water to the reaction mixture, pure precipitated products were obtained, filtered and washed with further water. In most cases, no further purification was necessary. All the product were known compounds which were identified by comparison of their melting points with those authentic literature samples (Table 1).

### Spectral data of the selected products

3,5-Dimethyl-4-phenyl-1,4,7,8-tetrahydrodipyrazolo[3,4-b:4′,3′-e]pyridine **(5a)**: IR (KBr: *ῡ* /cm^−1^): 3528, 3301, 2925, 1614, 1515, 1375; ^1^H NMR (300.13 MHz, DMSO-*d*_6_): δ = 2.06 (s, 6H, CH_3_), 4.81 (s, 1H, CH), 7.14–7.20 (m, 5H, H-Ar), 11.24–11.32 (br s, 3H, NH); ^13^C NMR (75.47 MHz, DMSO-*d*_6_): δ = 10.8, 33.1, 104.6, 125.8, 127.9, 128.1, 140.2, 143.7, 161.5.

4-(4-Chlorophenyl)-3,5-dimethyl-1,4,7,8-tetrahydrodipyrazolo[3,4-b:4′,3′-e]pyridine **(5f)**: IR (KBr,: *ῡ*/cm^−1^): 3365, 3100, 2854, 1612, 1526, 1368; ^1^H NMR (300.13 MHz, DMSO-*d*_6_): δ = 2.06 (s, 6H, CH_3_), 4.80 (s, 1H, CH), 7.12 (br s, 2H, H-Ar), 7.24 (br s, 2H, H-Ar), 11.35 (br s, 3H, NH); ^13^C NMR (75.47 MHz, DMSO-*d*_6_): δ = 10.7, 32.6, 104.3, 128.0, 129.8, 130.4, 140.1, 142.7, 161.4.

## Conclusions

In summary, a novel, environmentally-friendly, low-cost and reusable magnetic mesoporous HNTs were achieved via an efficient synthetic process at 500 °C. The structure, morphology, magnetic properties and thermal stability of the nanocomposite were assessed by using different analytical techniques. SEM and TEM images confirmed the stability and integrity of basic tube structure of HNTs and also, good dispersion of CuFe_2_O_4_ NPs. The porosity of CuFe_2_O_4_@HNTs nanocomposite was calculated by BET analysis and the loading of CuFe_2_O_4_ into and onto the HNTs were confirmed by pore volume and pore size information. XRD analysis approved the loading of CuFe_2_O_4_ NPs by the tetragonal structure. In addition, other unique features of the nanocomposite such as thermal stability and magnetic property were examined by TG and VSM analyses. To prove the catalytic ability of the present nanocomposite, it was used as an efficient and recyclable heterogeneous nanocatalyst in the synthesis of pyrazolopyridine derivatives via a four-component reaction. The products were obtained in high-to-excellent yields at room temperature under mild reaction conditions. The catalyst can be easily recovered and reused several times without significant activation loss by simple washing and drying. Furthermore, this is the first report on design, preparation, functionalization and characterization of the present nanocomposite and its performance as a heterogeneous catalyst in an important organic reaction.

## Supplementary information

Supplementary Information
