# Supplementary material for: Mesoporous halloysite nanotubes modified by CuFe2O4 spinel ferrite nanoparticles and study of its application as a novel and efficient heterogeneous catalyst in the synthesis of pyrazolopyridine derivatives
Source: Sci Rep. 2019 Apr 3;9:5552. doi: 10.1038/s41598-019-42126-9 (PMC6447565; doi:10.1038/s41598-019-42126-9)
Supplement: Supplementary file 1 — Supplementary Information [file 41598_2019_42126_MOESM1_ESM.doc]

**Mesoporous halloysite nanotubes modified by CuFe_2_O_4_ spinel ferrite nanoparticles and study of its application as a novel and efficient heterogeneous catalyst in the synthesis of pyrazolopyridine derivatives**

Ali Maleki,*^a^ Zoleikha Hajizadeh^a^*,* Peyman Salehi^b^

*^a^Catalysts and Organic Synthesis Research Laboratory, Department of Chemistry, Iran University of Science and Technology, Tehran 16846-13114, Iran*

*^b^Department of Phytochemistry, Medicinal Plants and Drugs Research Institute, Shahid Beheshti University, Evin, Tehran, Iran*

**Corresponding author. E-mail:* [*maleki@iust.ac.ir*](mailto:maleki@iust.ac.ir)

| **Table of contents** |
| --- |
| **Subject Page** |
| **Table S1.** Optimization of the reaction conditions in the synthesis of pyrazolopyridine **5a** ……………….S2 |
| **Figure S1.** ^1^H NMR spectrum of the product **5a** ……………………….……………..………………….S3 |
| **Figure S2.** ^13^C NMR spectrum of the product **5a**  …………………...………………………….….……..S4 |
| **Figure S3.** ^1^H NMR spectrum of the product **5f**  ………………………………………………..….……...S5  **Figure S4.** ^13^C spectrum of the product **5f** ………………………………………………………………S6 |
| **Figure S5.** Reusability of CuFe_2_O_4_@HNTs nanocatalyst in the synthesis of **5a** ……………..….….……..S7  **Figure S6.** FT-IR spectra of the recycled catalyst ………………………………………………..….……..S8  **Figure S7.** EDX analysis of the recycled catalyst ………………………………………………..….……..S9 |

**Table S1**. Optimizing of the reaction conditions in the synthesis of pyrazolopyridine **5a**.^a^

| Entry | Solvent | Catalyst | Catalyst amount (g) | Yield^a^ (%) |
| --- | --- | --- | --- | --- |
| 1 | H_2_O | - | - | Trace |
| 2 | EtOH | - | - | Trace |
| 2 | H_2_O | CuFe_2_O_4_@HNTs | 0.05 | 46 |
| 3 | EtOH | CuFe_2_O_4_ | 0.05 | 53 |
| 4 | EtOH | HNTs | 0.05 | 28 |
| 5 | EtOH | CuFe_2_O_4_@HNTs | 0.02 | 74 |
| 6 | EtOH | CuFe_2_O_4_@HNTs | 0.03 | 82 |
| 7 | EtOH | CuFe_2_O_4_@HNTs | 0.04 | 88 |
| 8 | EtOH | CuFe_2_O_4_@HNTs | 0.05 | 96 |
| 9 | EtOH | CuFe_2_O_4_@HNTs | 0.06 | 96 |
| 10 | EtOH | CuFe_2_O_4_@HNTs | 0.07 | 96 |

^a^ Reaction condition: hydrazine hydrate (2 mmol), ethyl acetoacetate (2 mmol), benzaldehyde (1 mmol), and ammonium acetate (3 mmol) in the presence of CuFe_2_O_4_@HNTs nanocomposite (0.05 g) at room temperature in the ethanol.

^b^ Isolated yield.


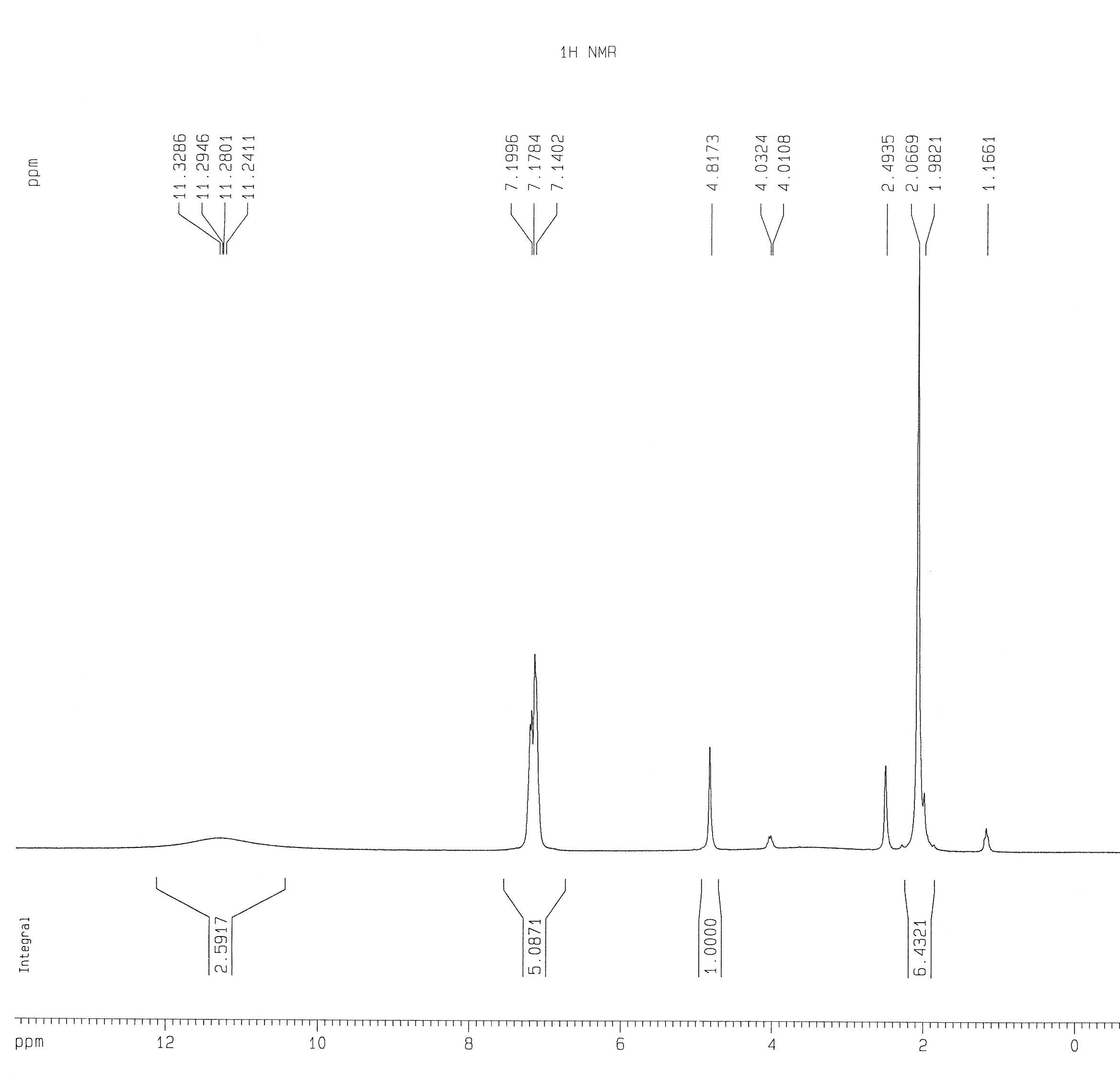


**Figure S1.** ^1^H NMR spectrum of the product **5a.**


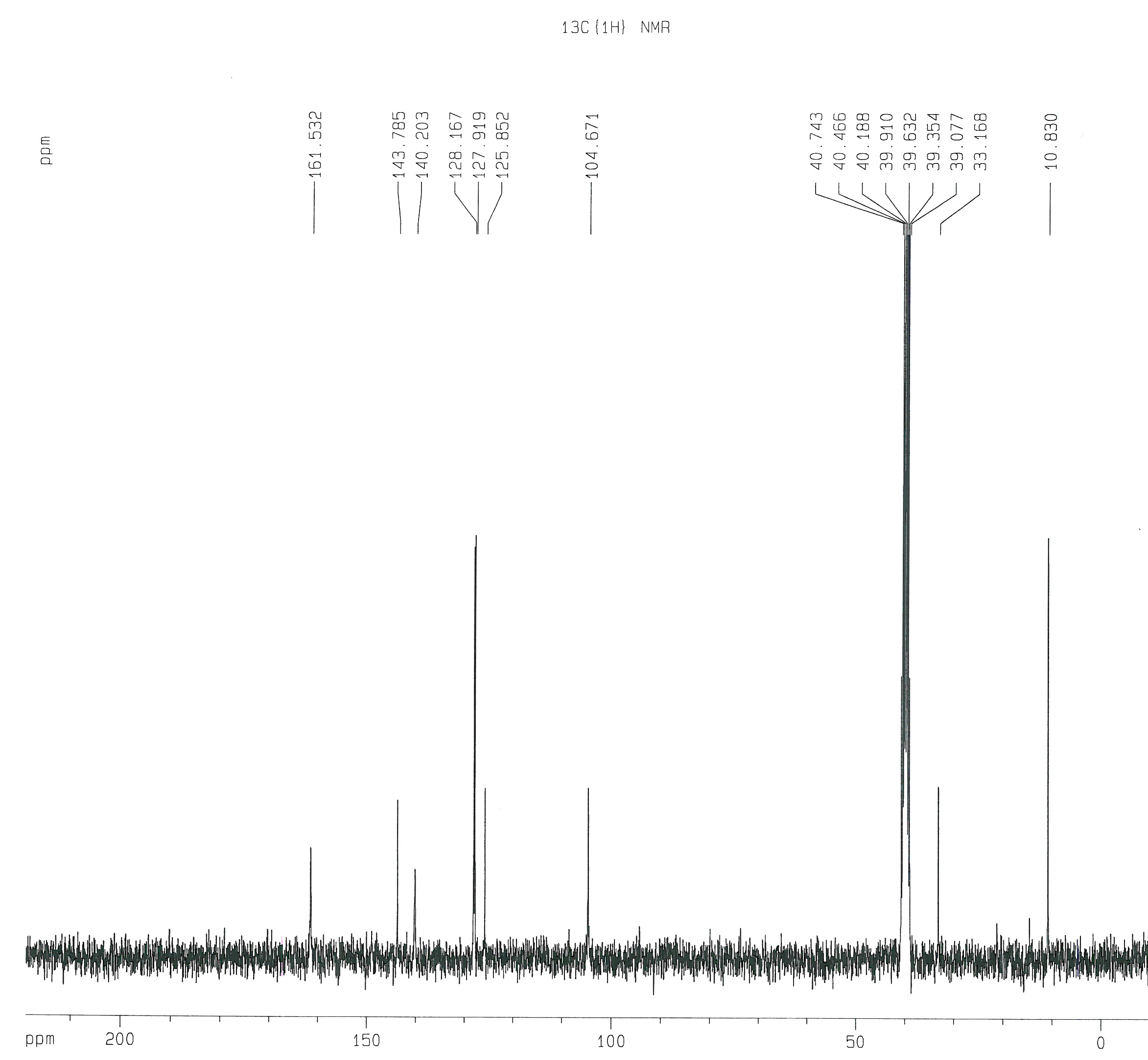


**Figure S2.** ^13^C NMR spectrum of the product **5a**.

**
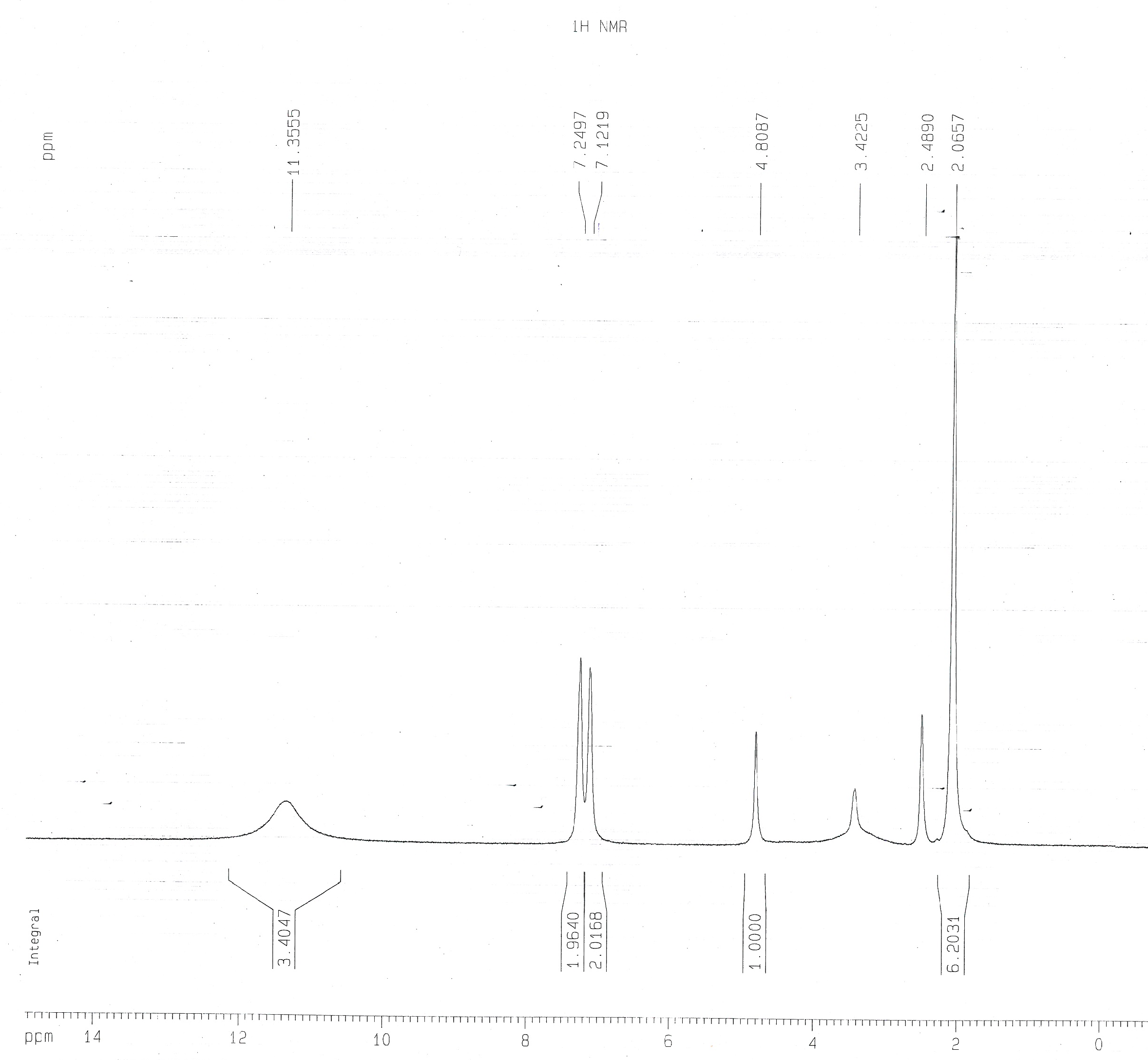
**

**Figure S3.** ^1^H NMR spectrum of the product **5f.**


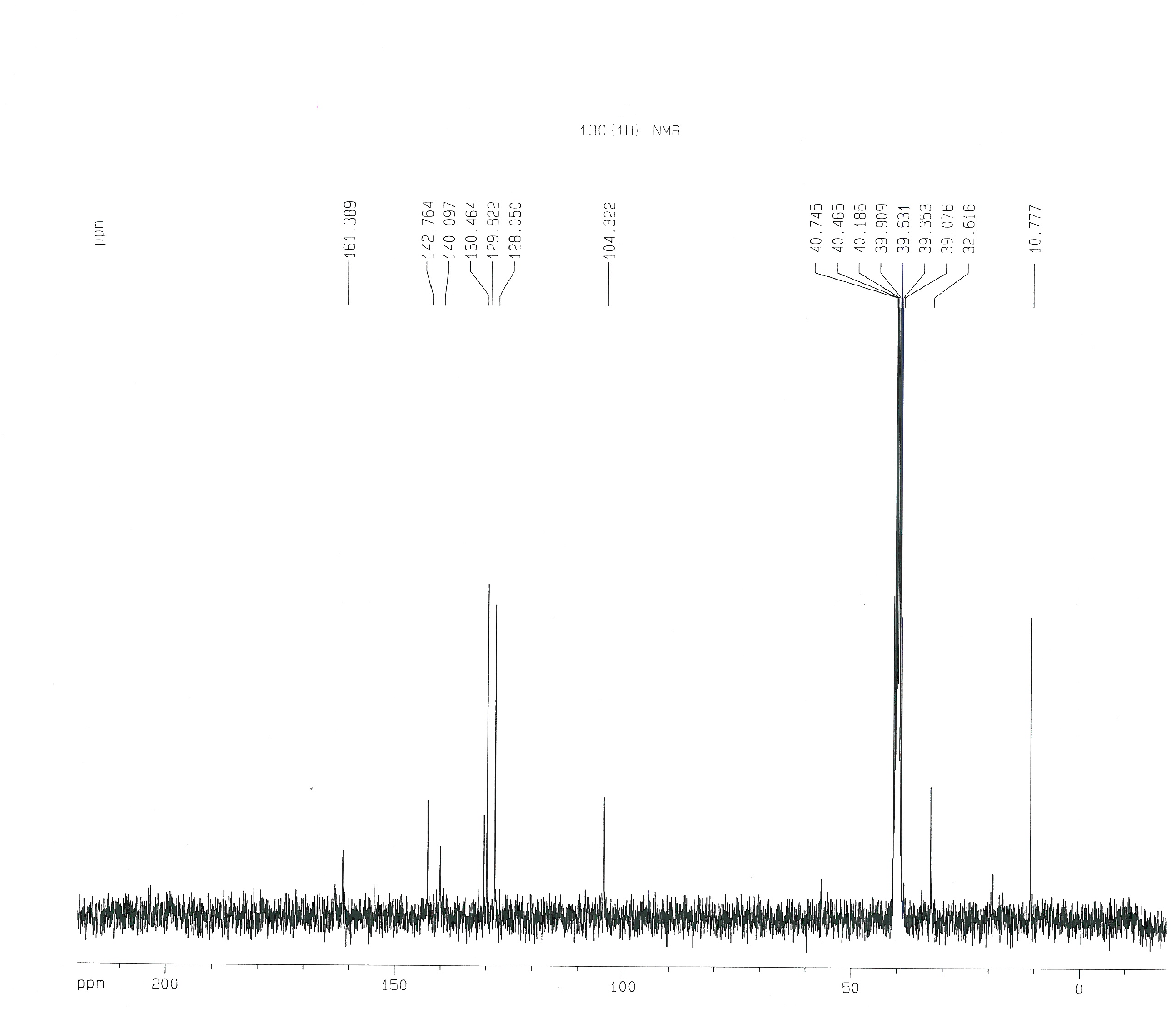


**Figure S4.** ^13^C NMR spectrum of the product **5f**.


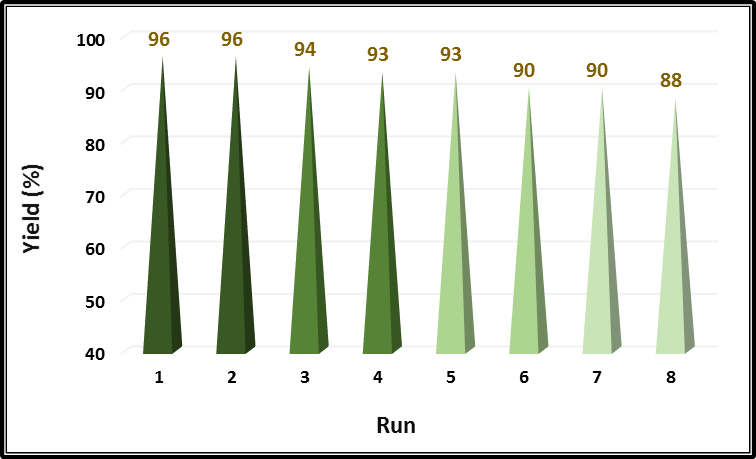


**Figure S5.** Reusability of CuFe_2_O_4_@HNTs nanocatalyst in the synthesis of **5a.**


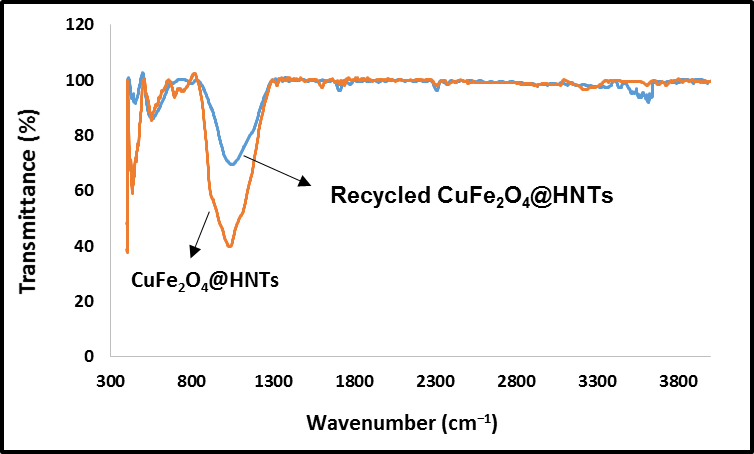


**Figure S6.** FT-IR spectra of the catalyst and recycled catalyst.


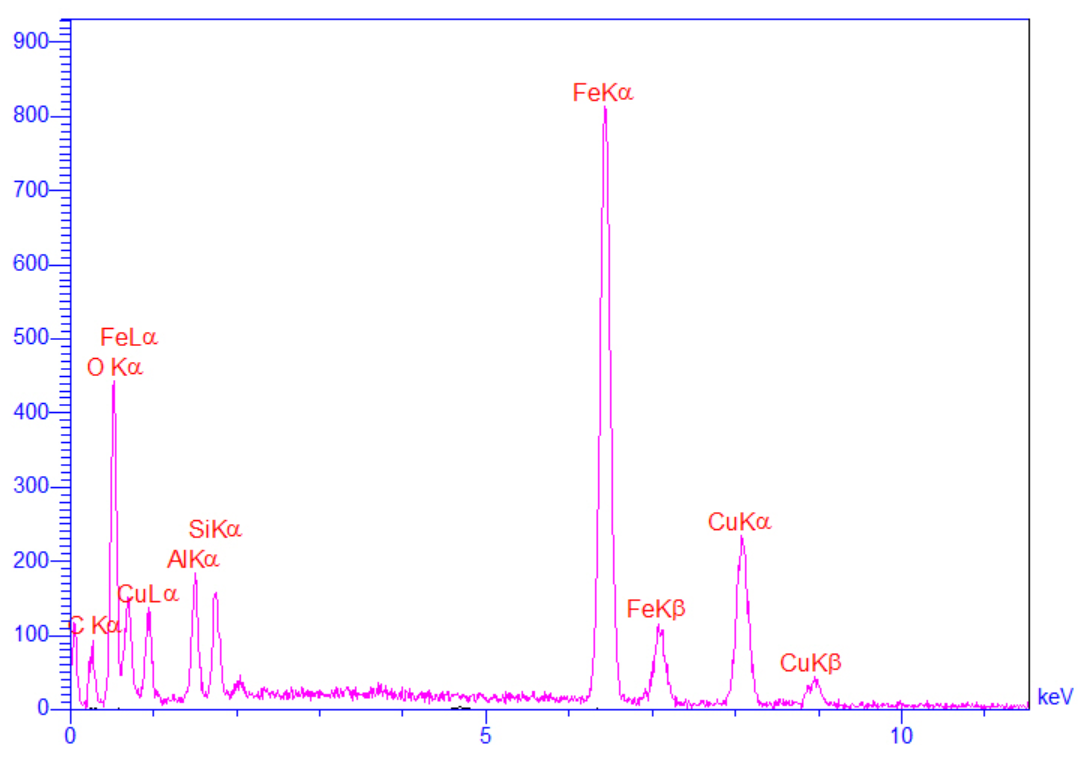


**Figure S7.** EDX analysis of the recycled catalyst.
